# Haemoglobin A1c and serum glucose levels and risk of gastric cancer: a systematic review and meta-analysis

**DOI:** 10.1038/s41416-021-01693-3

**Published:** 2022-01-13

**Authors:** Jiaojiao Zheng, Yunhe Gao, Shao-Hua Xie, Giola Santoni, Jesper Lagergren

**Affiliations:** 1 Upper Gastrointestinal Surgery, Department of Molecular Medicine and Surgery, Karolinska Institutet, Retzius Street 13a, 4th Floor, 171 77 Stockholm, Sweden; 2 Department of General Surgery, Zhongshan Hospital, Fudan University, No. 180 Fenglin Road, 200032 Shanghai, China; 3 Department of General Surgery, Chinese PLA General Hospital, No.28 Fuxing Road, Haidian District, 100853 Beijing, China; 4 School of Public Health and Key Laboratory of Ministry of Education for Gastrointestinal Cancer, Fujian Medical University, 350122 Fuzhou, China; 5 School of Cancer and Pharmaceutical Sciences, King’s College London and Guy’s and St Thomas’ NHS Foundation Trust, Guy’s Campus, Bermondsey Wing, 3rd Floor, London, SE1 9RT UK

**Keywords:** Gastric cancer, Risk factors, Diabetes, Epidemiology

## Abstract

**Background:**

This systematic review and meta-analysis examined associations between serum levels of haemoglobin A1c (HbA1c) and glucose and the risk of gastric cancer.

**Methods:**

MEDLINE, Embase, and Cochrane Library were searched for studies examining associations between serum levels of HbA1c or glucose and the risk of gastric cancer. Inclusion of studies, quality assessment, and data extraction were conducted independently by two authors. Pooled hazard ratios (HR) with 95% confidence intervals (CI) were synthesised using random-effects models. Cochran’s *Q* test and *I*^2^ statistic were used to assess heterogeneity.

**Results:**

Among 3473 identified studies, 12 were included. Of these, 5 studies examined HbA1c levels and 7 studies examined serum glucose levels. Serum HbA1c levels >6% were associated with an increased risk of gastric cancer (HR 1.36, 95% CI 1.06–1.74). When compared with the lowest glucose categories, the highest glucose categories were associated with a borderline increased risk of gastric cancer (HR 1.11, 95% CI 0.98–1.26). In subgroup analyses, studies that adjusted for *Helicobacter pylori* infection indicated stronger associations between elevated HbA1c levels and gastric cancer (HR 2.08, 95% CI 1.46–2.98) than those without such adjustment (HR 1.10, 95% CI 0.91–1.32).

**Conclusions:**

Long-standing poor glycaemic control may increase the risk of gastric cancer.

**Registration number:**

PROSPERO CRD42020157453.

## Introduction

Gastric cancer occurs in over one million individuals and causes nearly 800,000 deaths each year globally [1]. The incidence of gastric cancer is higher in East Asian countries than in Western countries [1]. The overall prognosis in gastric cancer is poor with a 5-year survival rate below 30% in most countries [2].

Diabetes mellitus is a chronic metabolic disease with increasing prevalence and is characterised by elevated serum levels of glucose and glycated haemoglobin A1c (HbA1c). Diabetes increases the risk of some tumours, but studies that have investigated diabetes in relation to the risk of developing gastric cancer have yielded conflicting results [3–6]. The inconsistency across these studies might, at least partly, be due to different methods of defining diabetes, ranging from diagnosis codes to use of anti-diabetes medications [4, 5, 7]. These differences may have introduced heterogeneity in the meta-analyses that pooled results from these studies [8–10]. In contrast, this systematic review and meta-analysis specifically investigated whether elevated levels of measured glucose biomarkers in serum, i.e. HbA1c and glucose, are associated with an increased risk of gastric cancer. These two biomarkers were chosen as they were the most common measurements for diabetes in clinical practice [11].

## Methods

### Search strategy and study selection

This systematic review and meta-analysis was performed and reported in accordance with the MOOSE guidelines [12]. The study protocol was registered on PROSPERO (CRD42020157453) before the systematic search was conducted. The literature search strategy (presented in detail in Supplementary Part 1) was discussed and agreed upon by all authors and was consulted with librarians at Karolinska Institutet. The systematic search for studies reporting associations between serum levels of HbA1c or glucose and the risk of gastric cancer was conducted in three databases: MEDLINE, Embase, and Cochrane Library. The original search was conducted on 15th December 2019, and was updated on 6th January 2021. No search restrictions were applied. In addition to the electronic search, reference lists of all included studies and relevant review articles were searched manually [8–10, 13–21]. Corresponding authors of three conference abstracts were contacted and one provided the full study [22]. Studies fulfilling the following criteria underwent full-text review: (a) original data on the associations between serum levels of HbA1c or glucose and the risk of gastric cancer; (b) cohort studies, case–control studies or randomised clinical trials where serum HbA1c or glucose levels were measured before the onset of gastric cancer; and (c) full-text reports available in English language. Studies reporting mortality from gastric cancer as a surrogate of incidence were excluded. Whenever multiple reports were based on the same study population, only the one with the largest sample size was included. However, if two studies from the same study population reported HbA1c and serum glucose separately, both studies were included. One author (JZ) retrieved the results from the databases and removed duplicates. The initial screening of study titles and abstracts, two rounds of full-text reviews, and the final inclusion of studies were performed independently by two authors (JZ and YG). Any disagreement was solved by consultation with a third author (SX).

### Quality assessment and data extraction

The quality of the included studies was independently assessed by two authors (JZ and YG) using a modified Newcastle-Ottawa Scale for cohort studies [23], where three sources of bias (selection bias, information bias, and confounding) were evaluated. The evaluation of each type of bias included 3–6 items and was summarised into low, moderate, or high risk of bias. The quality of the only included nested case–control study [4] was evaluated with reference to its source cohort [24]. A study assessed as no high risk in any of the three sources of bias and low risk in at least two sources of bias was defined as low risk of bias in general, whereas studies that did not meet these criteria were defined as having moderate to high risk of bias [25].

One author (JZ) recorded descriptive details for each included study, i.e. authorship, publication year, study design, setting, country, age and sex distribution of participants, total number of participants, study period, follow-up time, exposure (HbA1c or glucose), primary outcome, and number of incident gastric cancer cases. Relative risk estimates of the association, i.e. hazard ratios (HR), risk ratios or odds ratios and their 95% confidence intervals (CI) were independently extracted by two authors (JZ and YG). When multiple estimates were provided in the same study, we selected the estimates adjusted for four predefined potential confounders, i.e. sex, age, obesity and *Helicobacter pylori* infection. If the reported risk estimates were not adjusted for all these variables, the one adjusted for most of these confounders was used. If the results were reported separately for men and women in a study, both estimates were retrieved and treated as two separate results in the later analyses.

Any disagreement during the quality assessment or data extraction was solved together with a third author (SX).

### Statistical analysis

Associations between serum levels of HbA1c or glucose and the risk of gastric cancer were pooled using a random-effects model. Because all but one included study estimated HRs, and the remaining one estimated odds ratios, which were assumed to approximate HRs under the rare disease condition, HRs were used to synthesise the associations.

Studies reporting HbA1c had generally similar categorisations of HbA1c levels. The HRs were pooled comparing the HbA1c levels >6% with <6%, and also for the categories 6%-7% and >7%, both with the reference level <6%. Studies reporting serum glucose levels had different cut-offs. Therefore, we pooled the HRs comparing the highest category with the lowest category. Five studies of serum glucose levels used quartile or quintile cut-off values, [4, 22, 26–28] and the retrieved HRs were converted into estimates comparing the highest with the lowest tertiles to minimise heterogeneity across studies according to the method reported by Genevieve et al. [29]. Where appropriate, HRs from studies reporting fasting glucose were also pooled into the categories 6–7 mmol/L and >7 mmol/L, both with the reference level <6 mmol/L. The pooling of HRs was performed according to the method reported by Tierney et al. [30], using the generic inverse-variance method proposed by the Cochrane Handbook [31].

Heterogeneity across studies was assessed by Cochran’s *Q* test and *I*^2^ statistics. A *P* value <0.1 in the Cochran’s *Q* test was considered statistically significant [31]. An *I*^2^ value ≤25% represented low heterogeneity, 25–49% moderate heterogeneity, and ≥50% high heterogeneity [32]. To explore potential sources of heterogeneity, subgroup analyses were conducted by sex (female or male), geographic area (Asian or non-Asian countries), risk of bias (moderate to high or low), adjustment for obesity (yes or no), and adjustment for *Helicobacter pylori* infection (yes or no). In a sensitivity analysis of serum glucose, the only case–control study was excluded [4]. Because none of the included studies specified whether it was type I or type II diabetes in participants with hyperglycaemia, type I and type II diabetes were not analysed separately.

Publication bias was evaluated by the visual inspection of funnel plots, Egger’s test, and a nonparametric trim-and-fill analysis.

All analyses were conducted using Stata 16 (StataCorp, College Station, Texas, USA). All statistical tests were two-sided.

## Results

### Literature search and study characteristics

**Fig. 1:**
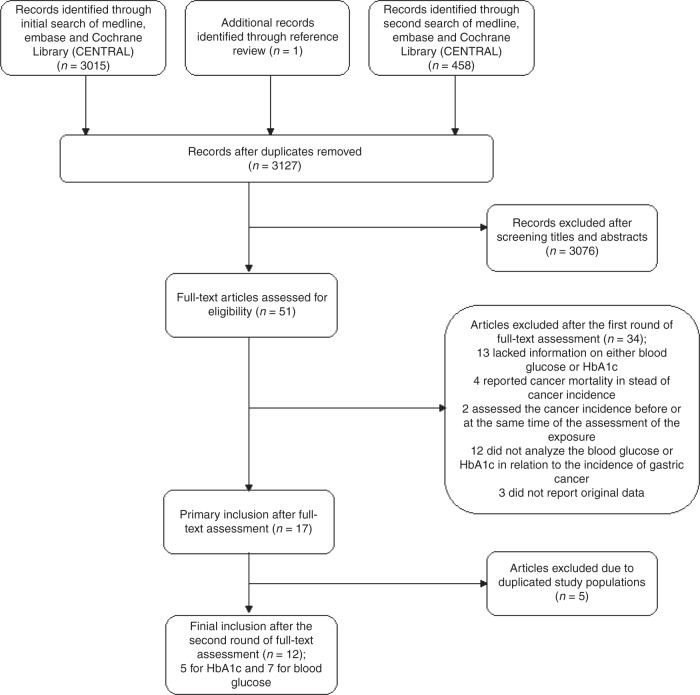
Flowchart of study selection.

The search and selection of studies are presented in a flowchart (Fig. 1). Among 3473 publications identified from the electronic databases and reference list search, 12 studies met the inclusion criteria. Of these studies, 5 reported serum HbA1c levels [33–37], and 7 investigated serum glucose levels. [4, 22, 26–28, 38, 39] Among the studies reporting serum glucose levels, 4 analysed fasting samples [22, 26, 28, 38], 2 analysed random samples [4, 39], and the remaining study analysed both [27]. Among all included studies, 11 were cohort studies [22, 26–28, 33–39] and one was a nested case–control study [4]. Eight studies were conducted in Asia, [4, 26, 28, 34–36, 38, 39] 3 in Europe [22, 27, 37], and 1 in New Zealand [33]. Nine studies were based on screening programmes, surveys, or routine health check-ups. The study period of the included studies ranged from 1972 to 2016. Two studies were based on the same study population, but reported serum levels of HbA1c and glucose in relation to risk of gastric cancer in separate publications [26, 34]. All studies included both sexes, and 3 reported sex-specific risk estimates [4, 27, 38]. Six studies analysed diabetes biomarkers in relation to the risk of multiple cancer types, including gastric cancer, [22, 33, 35, 37–39] while the other 6 investigated gastric cancer risk only, [4, 26–28, 34, 36] including 2 that specifically studied gastric adenocarcinoma (Table 1) [27, 28].

**Table 1:** Summary of characteristics of studies included in the meta-analysis.

Biomarker	First author and publication year	Study design	Settings	Country	Number of participants	Male (%)	Age distribution (years)	Study period (mean/median follow-up in years)	Primary outcome	Number of gastric cancer cases	Overall risk of bias^a^
HbA1c^b^	Travier 2007	Cohort	Screening	New Zealand	46,575	44.6	>= 18, and more than 60% <40	1999–2004 (4.4)	All cancer incidence	25	Moderate to high
HbA1c	Ikeda 2009	Cohort	Screening	Japan	2603	41.2	Mean age 59.2	1988–2002 (not reported)	Gastric cancer incidence	97	Low
HbA1c	Goto 2016	Cohort	Health check-up	Japan	29,629	38.3	Mean age 62.6	1998–2008 (8.5)	All cancer incidence	282	Low
HbA1c	Cheung 2019	Cohort	Registry-based cohort	China (Hong Kong)	7266	52.0	Median age 65.2 (interquartile range 56.1–74.2)	2003–2015 (5.5)	Gastric cancer incidence	37	Low
HbA1c	Peila 2020	Cohort	Biobank cohort	United Kingdom	476,517	45.6	Mean age 56.4	2006–2016 (7.1)	All cancer incidence	380	Moderate to high
Serum glucose	Jee 2005	Cohort	Health check-up	Korea	1,298,385	63.9	Mean age for men 45.3; mean age for women 49.6	1992–2002 (not reported)	All cancer incidence	Not reported	Moderate to high
Serum glucose	Yamagata 2005	Cohort	Screening	Japan	2466	41.7	Mean age for men 57.5; mean age for women 58.8	1988–1997 (not reported)	Gastric cancer incidence	66	Low
Serum glucose	Wulaningsih 2012	Cohort	Screening	Sweden	540,309	53.7	Mean age for participants with gastric cancer 57.8	1985–2002 (12)	Incidence of cancer of the oesophagus, stomach, colon, and rectum	776	Moderate to high
Serum glucose (fasting and non-fasting)	Lindkvist 2013	Cohort	Registry-based cohorts	Norway, Austria, Sweden	578,700	50.1	Mean age for men 43.9; mean age for women 44.1	Norwegian cohorts 1972–2003; Austrian cohort 1985–2003; Swedish cohorts 1985–2006 (10.4)	Gastric adenocarcinoma incidence	1210	Moderate to high
Serum glucose (non-fasting)	Hidaka 2015	Nested case–control	Screening	Japan	123,576	66.9	Mean age 57.2	1990–2004 (not applicable)	Gastric cancer incidence	477	Low
Serum glucose	Kim 2016	Cohort	Health check-up	Korea	23,218	62.4	Mean age for participants with gastric cancer 56.5; mean age for participants without gastric cancer 53.2	2004–2015 (6.8)	Gastric adenocarcinoma incidence	154	Moderate to high
Serum glucose (non-fasting)	Pan 2018	Cohort	Prospective cohorts	China	508,892	41.0	Age range 30-79; more than 75% between 30-59	2004–2013 (7.1)	All cancer incidence	2104	Moderate to high

^a^Assessed by the Newcastle-Ottawa Scale, for which the details are available in Supplementary Table 1.

^b^Haemoglobin A1c.

### Quality assessment

The overall quality of included studies in terms of risk of bias is summarised in Table 1, and a more detailed assessment is provided in the Supplementary Table 1. Five studies had low overall risk of bias and the other 7 had moderate to high risk of bias. More specially, 6 studies had moderate to high risk of selection bias, 6 studies had moderate to high risk of information bias, and 8 studies had moderate to high risk of bias due to confounding. All 12 studies adjusted for sex and age in their main analyses, 8 adjusted for obesity, and 4 adjusted for *Helicobacter pylori* infection. In addition, 11 studies adjusted for tobacco smoking (Supplementary Table 2).

### HbA1c levels and risk of gastric cancer

**Fig. 2:**
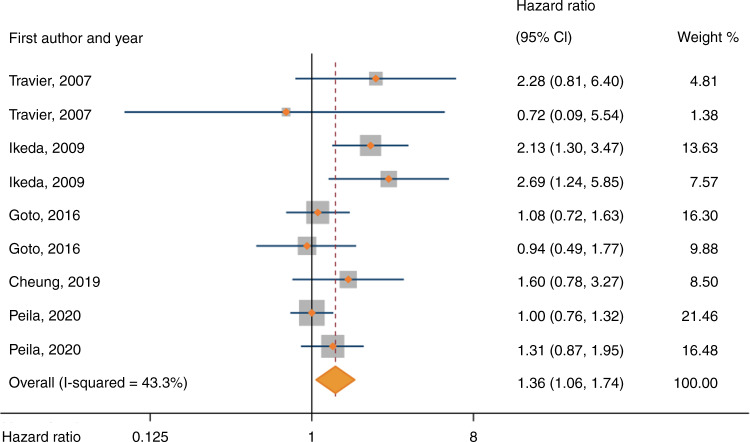
Forest plot of risk estimates for associations between serum haemoglobin A1c levels and risk of gastric cancer under the random-effects model. The studies are ordered chronologically. CI: confidence interval.

The meta-analysis of HbA1c included 562,590 participants from 5 studies, of whom 821 (0.1%) developed gastric cancer during follow-up. Random-effects meta-analysis showed that HbA1c > 6% were associated with an increased risk of gastric cancer (pooled HR 1.36, 95% CI 1.06-1.74) (Fig. 2). When pooling HRs by the three cut-offs of HbA1c, the pooled HRs were 1.36 (95% CI 0.91–2.02) for the cut-off 6–7% vs < 6%, and 1.39 (95% CI 1.00–1.94) for the cut-off >7% vs < 6%) (as shown in Supplementary Fig. 1). There was moderate heterogeneity across these studies (*I*^2^ = 43%, *P* in Q test = 0.43). Subgroup analysis showed that adjustment for *Helicobacter pylori* infection was a source of heterogeneity among studies (*P* for group difference = 0.002), and the association between elevated HbA1c levels and risk of gastric cancer was stronger in studies with adjustment for *Helicobacter pylori* infection (pooled HR 2.08, 95% CI 1.46–2.98) than those without such adjustment (pooled HR 1.10, 95% CI 0.91–1.32) (Table 2). Subgroup analyses by sex, geographic area, risk of bias, or adjustment for obesity did not reveal any of these factors as sources of heterogeneity (Table 2).

**Table 2:** Subgroup meta-analyses for elevated Haemoglobin A1c and serum glucose levels in relation to risk of gastric cancer.

	Haemoglobin A1c					Serum glucose				
Study characteristics	Number of studies	Pooled HR (95% CI)	*P*_Heterogeneity_^a^	*P*_Difference_^b^	*I*^2^ (%)	Number of studies	Pooled HR (95% CI)	*P*_Heterogeneity_^a^	*P*_Difference_^b^	*I*^2^ (%)
Geographical area										
Asia	3	1.51 (1.03–2.21)	0.07	0.13	54	5	1.14 (0.96–1.35)	0.13	0.73	75
Non-Asia	2	1.14 (0.89–1.45)	0.36		7	2	1.08 (0.86–1.36)	0.49		71
Sex										
Male	2	1.08 (0.86–1.36)	0.55	0.92	0	3	0.99 (0.92–1.08)	0.94	0.64	0
Female	2	1.10 (0.71–1.69)	0.24		29.5	3	1.08 (0.75–1.56)	0.02		74
Assessment of risk of bias										
Moderate to high	2	1.14 (0.89–1.45)	0.36	0.13	7	5	1.09 (0.98–1.22)	0.01	0.58	88
Low	3	1.51 (1.03–2.21)	0.07		54	2	1.57 (0.44–5.63)	<0.01		66
Adjustment for *Helicobacter pylori* infection										
Yes	2	2.08 (1.46–2.98)	0.63	<0.01	0	2	1.57 (0.44–5.63)	<0.01	0.58	66
No	3	1.10 (0.91–1.32)	0.62		0	5	1.09 (0.98–1.22)	0.01		88
Adjustment for obesity										
Yes	3	1.32 (0.99–1.76)	0.03	0.30	58.4	5	1.16 (0.96–1.42)	<0.01	0.23	75
No	2	1.68 (0.95–2.95)	0.61		0	2	1.02 (0.93–1.11)	0.32		12

*HR* hazard ratio, *CI* confidence interval.

^a^*P* value in Cochran’s *Q* test.

^b^*P* value in the test of subgroup difference.

### Serum glucose levels and risk of gastric cancer

**Fig. 3:**
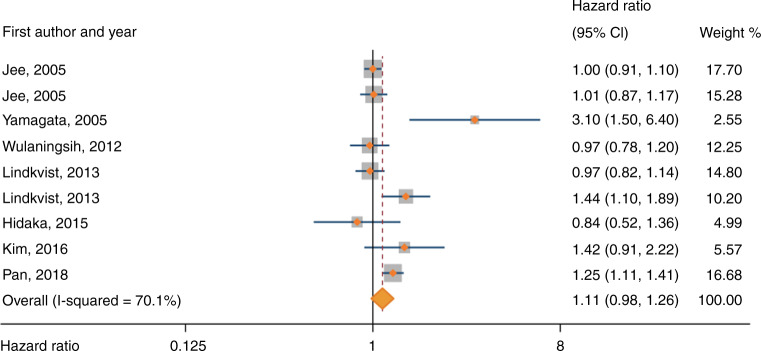
Forest plot of risk estimates for associations between serum glucose levels and risk of gastric cancer under the random-effects model. The studies are ordered chronologically. CI: confidence interval.

The meta-analysis of serum glucose included 3,075,546 participants from 10 studies. Of these, 4787 (0.2%) participants developed gastric cancer, but the number of participants developing gastric cancer was missing in one study [38]. Random-effects meta-analysis showed a borderline increased risk of gastric cancer associated with the highest categories of serum glucose compared with lowest categories (pooled HR 1.11, 95% CI 0.98–1.26) (Fig. 3). There was high heterogeneity (*I*^2^ = 70%, *P* in Q test = 0.001) across studies. The pooled HRs between levels of fasting glucose and risk of gastric cancer were 1.16 (95% CI 0.86–1.55) for the cut-off 6–7 mmol/L vs < 6 mmol/L, and 1.00 (95% CI 0.89–1.13) for the cut-off >7 mmol/L vs < 6 mmol/L (as shown in Supplementary Fig. 2). Subgroup analyses by sex, geographic area, risk of bias, adjustment for obesity, or adjustment for *Helicobacter pylori* infection did not reveal any of these factors as sources of heterogeneity (Table 2). The sensitivity analysis excluding the nested case–control study yielded a similar risk estimate as that of the main analysis (pooled HR 1.13, 95% CI 0.99–1.28).

### Publication bias

**Fig. 4:**
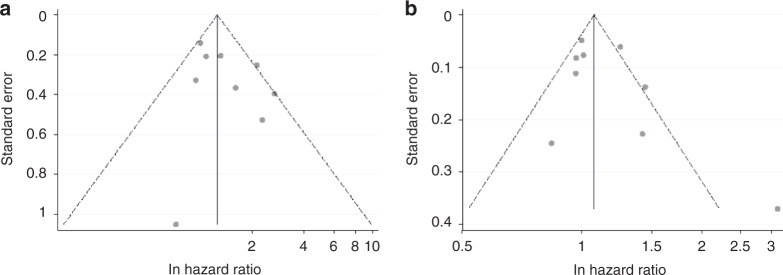
Funnel plots for studies of serum haemoglobin A1c (a) and glucose levels (b). Each solid dot represents one effects size retrieved from the original study.

Asymmetry was suggested for studies reporting serum HbA1c and glucose in the funnel plots (Fig. 4). The Egger’s tests showed no evidence of publication bias for studies reporting HbA1c (*P* = 0.292), but for studies reporting serum glucose (*P* = 0.055). When imputing potential missing studies with trim-and-fill analysis, the pooled HR for serum glucose became slightly attenuated (pooled HR 1.05, 95% CI 0.89–1.24).

## Discussion

This is the first systematic review and meta-analysis examining measured serum levels of HbA1c and glucose in relation to the risk of developing gastric cancer. The results indicate that long-standing poor glycaemic control, as indicated by elevated HbA1c levels, increases the risk of gastric cancer, while the association between elevated serum glucose levels and gastric cancer is less clear.

Strengths of this study include an a priori defined protocol, broad and systematic search strategy, and assessment of different sources of bias. There are also limitations. First, the number of studies was limited and some important information, e.g. history of medication use and the diagnosis of diabetes, were not reported in most individual studies, reducing the possibility and statistical power for more subgroup analyses. Second, the cut-off values of serum HbA1c and glucose were not completely in accordance with the clinical definitions of diabetes and prediabetes. Third, there was high heterogeneity across studies evaluating serum glucose that were not explained by differences in population characteristics or potential biases [40]. Forth, publication bias for studies investigating serum glucose levels could not be ruled out.

Possible mechanisms for hyperglycaemia leading to gastric cancer include promotion of cancer cell proliferation [41], induction of gastric mucosa atrophy [42], and influence on the insulin/insulin-like growth factors axis, which regulates proliferation, invasion, and apoptosis of gastric cancer cells [43, 44]. A potential explanation for the association between HbA1c levels, rather than glucose levels, and risk of gastric cancer might be that the carcinogenic influence of hyperglycaemia depends on the duration of it. While a single serum glucose test represents only a transitory status of blood glucose which is subject to many factors, e.g. fasting time and anti-diabetes medication, HbA1c levels reflect the average glucose levels during the past 2–3 months, and time-average HbA1c levels, which take into account of serial HbA1c measurements, reflect glycaemic status for even longer periods. A previous study suggested an association between diabetes and risk of gastric cancer only at least 5 years after the diabetes diagnosis [7]. Another study found that only long-lastingly elevated serum glucose levels (but not other trajectory patterns) were associated with an increased risk of gastrointestinal cancer (HR 1.66, 95% CI 1.22–2.26) [45]. Another potential link between high HbA1c levels and gastric carcinogenesis might be the treatment for long-term poor glycaemic control. Among many anti-diabetes medications, metformin (the first-line oral medication for diabetes) may decrease the risk of gastric cancer, while insulin might increase this risk [46, 47].

Although no previous systematic reviews and meta-analyses have examined diabetes biomarkers and the specific risk of developing gastric cancer, one study has suggested an increasing log-linear trend between HbA1c levels and gastric cancer [21]. Another meta-analysis of prediabetic levels of serum glucose suggested an increased risk of mortality from combined gastric and colorectal cancers (pooled relative risk 1.55, 95% CI 1.15–2.09) [16]. However, that study assessed cancer mortality rather than incidence and did not separately analyse gastric cancer.

Nevertheless, several systematic reviews and meta-analyses have evaluated diabetes (assessed by different methods) in relation to gastric cancer risk [8–10, 14, 17]. Two systematic review and meta-analyses found a positive association [9, 17], two suggested a positive association of borderline statistical significance [10, 14], and the most recent one found no association [8]. None of these meta-analyses accounted for the different methods of assessing diabetes in individual studies, which ranged widely from self-reported diabetes, diagnosis codes identified from medical records, data from healthcare registries or health insurances, use of anti-diabetic medication, to objectively measured diabetes biomarkers [4, 5, 7, 34, 48]. These differences may explain the high heterogeneity (*I*^2^ ranging from 70 to 95%) in the previous meta-analyses. The strict inclusion of only studies that examined objectively measured serum HbA1c in the present study counteracted the heterogeneity in the analysis of HbA1c. The remaining high heterogeneity in the analysis of serum glucose may be due to different cut-offs for glucose levels in individual studies as well as the mix of fasting and non-fasting serum glucose levels. This explains the decreased heterogeneity when pooling the data for fasting serum glucose only.

Some meta-analyses that examined diabetes and risk of gastric cancer indicated stronger associations among women [9, 10, 17] and East Asians [9, 14], which, however, was inconsistent with other meta-analyses [8, 10, 14, 17]. In the present study, subgroup analyses found no clear differences by sex, but a seemly stronger association between HbA1c and risk of gastric cancer in the Asian populations than in the non-Asian populations was indicated, although the difference between the two groups was not statistically significant. The present study also showed a stronger association between elevated HbA1c and gastric cancer in studies adjusting for *Helicobacter pylori* infection. *Helicobacter pylori* infection is the strongest risk factor for gastric cancer. Experimental studies have suggested that *Helicobacter pylori* infection might lead to hyperglycaemia, and thus confound the association between hyperglycaemia and risk of gastric cancer [49]. On the other hand, hyperglycaemia might stimulate *Helicobacter pylori* infection via various mechanisms, including enhancing its proliferation, viability, adhesion and CagA-phosphorylation [50]. Therefore, *Helicobacter pylori* infection might also act as a mediator in the development of gastric cancer associated with hyperglycaemia. Stratified analysis by *Helicobacter pylori* infection status and mediation analysis are warranted to clarify the interaction of hyperglycaemia and *Helicobacter pylori* infection in gastric cancer in the future.

The increased risk of gastric cancer among participants with high levels of HbA1c found in the present study indicates that individuals with poor glycaemic control for long periods may be a risk group for gastric cancer. However, given the limited number of studies published, more evidence is needed to confirm this association. More importantly, as most included studies in this review analysed multiple cancer sites, studies specifically analysing gastric cancer are needed to conduct meaningful subgroup analyses by sub-locations and known risk factors of gastric cancer. Last, any interactions between elevated levels of HbA1c and other factors, e.g. sex, ethnicity, diabetes treatment, and use of other medications such as statins or aspirin, in the development of gastric cancer are not clear and evidence from more large prospective studies is needed.

To conclude, this systematic review and meta-analysis indicates that long-term hyperglycaemia, as measured by HbA1c, increases the risk of gastric cancer.

## Supplementary information

Supplementary Material

Reproducibility checklist

## Data Availability

The full texts of all included studies were retrieved from the online databases Embase and MEDLINE. The data of this systematic review and meta-analyses are all pubic and available from Embase and Medline.
