# Correction: Haemoglobin A1c and serum glucose levels and risk of gastric cancer: a systematic review and meta-analysis

**DOI:** 10.1038/s41416-022-01761-2

**Published:** 2022-02-24

**Authors:** Jiaojiao Zheng, Yunhe Gao, Shao-Hua Xie, Giola Santoni, Jesper Lagergren

**Affiliations:** 1 Upper Gastrointestinal Surgery, Department of Molecular Medicine and Surgery, Karolinska Institutet, Retzius Street 13a, 4th Floor, 171 77 Stockholm, Sweden; 2 Department of General Surgery, Zhongshan Hospital, Fudan University, No. 180 Fenglin Road, 200032 Shanghai, China; 3 Department of General Surgery, Chinese PLA General Hospital, No.28 Fuxing Road, Haidian District, 100853 Beijing, China; 4 School of Public Health and Key Laboratory of Ministry of Education for Gastrointestinal Cancer, Fujian Medical University, 350122 Fuzhou, China; 5 School of Cancer and Pharmaceutical Sciences, King’s College London and Guy’s and St Thomas’ NHS Foundation Trust, Guy’s Campus, Bermondsey Wing, 3rd Floor, London, SE1 9RT UK

**Keywords:** Gastric cancer, Risk factors, Diabetes, Epidemiology

Correction to: *British Journal of Cancer* 10.1038/s41416-021-01693-3, published online 13 January 2022

The article “Haemoglobin A1c and serum glucose levels and risk of gastric cancer: a systematic review and meta-analysis”, written by Jiaojiao Zheng, Yunhe Gao, Shao-Hua Xie, Giola Santoni, Jesper Lagergren, was originally published electronically on the publisher’s internet portal on 13 January 2022 without open access. With the author(s)’ decision to opt for Open Choice the copyright of the article changed on 15 February 2022 to © The Author(s) 2022 and the article is forthwith distributed under a Creative Commons Attribution 4.0 International License, which permits use, sharing, adaptation, distribution and reproduction in any medium or format, as long as you give appropriate credit to the original author(s) and the source, provide a link to the Creative Commons licence, and indicate if changes were made. The images or other third party material in this article are included in the article’s Creative Commons licence, unless indicated otherwise in a credit line to the material. If material is not included in the article’s Creative Commons licence and your intended use is not permitted by statutory regulation or exceeds the permitted use, you will need to obtain permission directly from the copyright holder. To view a copy of this licence, visit http://creativecommons.org/licenses/by/4.0/.

Open access funding provided by Karolinska Institute.

