# Supplementary material for: Haemoglobin A1c and serum glucose levels and risk of gastric cancer: a systematic review and meta-analysis
Source: Br J Cancer. 2022 Jan 13;126(7):1100–7. doi: 10.1038/s41416-021-01693-3 (PMC8979989; doi:10.1038/s41416-021-01693-3)

**Part 1: Search Strategy**

Database: Medline

Combination:

1. exp Blood Glucose/ or Glucose Tolerance Test/

2. (((blood or fasting or post-load or postprandial or random or serum or plasma or test*) adj3 glucose*) or ((blood or serum or plasma) adj3 sugar)).ti,ab,kf.

3. ((diabet* adj3 biomarker*) or (glucose adj3 (disorder* or intolerance*)) or (impair* adj3 glucose)).ti,ab,kf.

4. exp Metabolic Syndrome/

5. (metabolic syndrome* or insulin resistan*).ti,ab,kf.

6. exp Hyperglycemia/

7. (hyperglycaemia* or hyperglycemia* or dysglycemia* or dysglycaemia* or glycemia* or glycaemia* or hyperglucemia* or hyperglucaemia* or hyperglycemic syndrome*).ti,ab,kf.

8. Prediabetic State/

9. (prediabet* or pre diabet*).ti,ab,kf.

10. exp Glycated Hemoglobin A/

11. (HbA1* or Hb A1* or hb a or hba 1c or hemoglobin* a or haemoglobin* a or ((hemoglobin or haemoglobin) adj1 A?1*) or glycohemoglobin* A* or glycohaemoglobin* A* or ((glycated or glycosylated) adj1 (hemoglobin* or haemoglobin*))).ti,ab,kf.

12. 1 or 2 or 3 or 4 or 5 or 6 or 7 or 8 or 9 or 10 or 11

13. exp Neoplasms/ep or Stomach Neoplasms/

14. ((gastric* or stomach* or gastrointestinal or digestive) adj3 (carcinoma* or neoplasm* or tumor* or tumour* or cancer* or malignan*)).ti,ab,kf.

15. 13 or 14

16. ((neoplasm* or tumor* or tumour* or cancer* or malignan*) adj3 (incidence or screening or detection or risk* or risk assessment* or risk factor*)).ti,ab,kf.

17. Risk/ or exp Risk Assessment/ or Risk Factors/

18. 16 or 17

19. 12 and 15 and 18

Database: Embase

Combination:

1. 'glucose blood level'/exp OR 'glucose blood level' OR 'glucose tolerance test’/exp OR ‘glucose tolerance test’

2. (((blood OR fasting OR post-load OR postprandial OR random OR serum OR plasma OR test*) NEAR/3 glucose*) OR ((blood OR serum OR plasma) NEAR/3 sugar)):ti,ab,kw

3. ((diabet* NEAR/3 biomarker*) OR (glucose NEAR/3 (disorder* OR intolerance*)) OR (impair* NEAR/3 glucose)):ti,ab,kw

4. 'metabolic syndrome x'/exp OR 'metabolic syndrome x'

5. (‘metabolic syndrome*’ OR ‘insulin resistan*’):ti,ab,kw

6. 'hyperglycemia'/exp OR 'hyperglycemia' OR 'dysglycemia'/exp OR 'dysglycemia'

7. (hyperglycaemia* OR hyperglycemia* OR dysglycemia* OR dysglycaemia* OR glycemia* OR glycaemia* OR hyperglucemia* OR hyperglucaemia* OR ‘hyperglycemic syndrome*’):ti,ab,kw

8. 'impaired glucose tolerance'/exp OR 'impaired glucose tolerance'

9. (prediabet* OR ‘pre diabet*’):ti,ab,kw

10. 'hemoglobin a1c'/exp OR 'hemoglobin a1c'

11. (HbA1* OR ‘Hb A1*’ OR ‘hb a’ OR ‘hba 1c’ OR ‘hemoglobin* a’ OR ‘haemoglobin* a’ OR ((hemoglobin OR haemoglobin) NEXT/1 A$1*) OR ‘glycohemoglobin* A*’ OR ‘glycohAemoglobin* A*’ OR ((glycated OR glycosylated) NEXT/1 (hemoglobin* OR haemoglobin*))):ti,ab,kw

12. #1 OR #2 OR #3 OR #4 OR #5 OR #6 OR #7 OR #8 OR #9 OR #10 OR #11

13. ('malignant neoplasms subdivided by anatomical site'/exp/dm_ep,dm_et OR 'malignant neoplasms subdivided by anatomical site') AND [humans]/lim AND [clinical study]/lim

14. 'stomach cancer'/exp OR 'stomach cancer'

15. ((gastric* OR stomach* OR gastrointestinal OR digestive) NEAR/3 (carcinoma* OR neoplasm* OR tumor* OR tumour* OR cancer* OR malignan*)):ti,ab,kw

16. #13 OR #14 OR #15

17. ((neoplasm* OR tumor* OR tumour* OR cancer* OR malignan*) NEAR/3 (incidence OR screening OR detection OR risk* OR ‘risk assessment*’ OR ‘risk factor*’)):ti,ab,kw

18. ('cancer risk'/exp OR 'cancer risk' OR 'risk assessment'/exp OR 'risk assessment' OR 'risk factor'/exp OR 'risk factor' OR 'cancer incidence'/exp OR 'cancer incidence')

19. #17 OR #18

20. #12 AND #16 AND #19

Database: Cochrane Library

Combination:

1. [mh “Blood Glucose”] OR [mh “Glucose Tolerance Test”]

2. (((blood OR fasting OR post-load OR postprandial OR random OR serum OR plasma OR test*) NEAR/3 glucose*) OR ((blood OR serum OR plasma) NEAR/3 sugar)):ti,ab,kw

3. ((diabet* NEAR/3 biomarker*) OR (glucose NEAR/3 (disorder* OR intolerance*)) OR (impair* NEAR/3 glucose)):ti,ab,kw

4. [mh “Metabolic Syndrome”]

5. ((metabolic NEXT syndrome*) OR (insulin NEXT resistan*)):ti,ab,kw

6. [mh Hyperglycemia]

7. (hyperglycaemia* OR hyperglycemia* OR dysglycemia* OR dysglycaemia* OR glycemia* OR glycaemia* OR hyperglucemia* OR hyperglucaemia* OR (hyperglycemic NEXT syndrome*)):ti,ab,kw

8. [mh “Prediabetic State”]

9. (prediabet* OR (pre NEXT diabet*)):ti,ab,kw

10. [mh “Glycated Hemoglobin A”]

11. (HbA1* OR (Hb NEXT A1*) OR “hb a” OR “hba 1c” OR (hemoglobin* NEXT a) OR (haemoglobin* NEXT a) OR ((hemoglobin OR haemoglobin) NEXT A?1*) OR (glycohemoglobin* NEXT A*) OR (glycohaemoglobin* NEXT A*) OR ((glycated or glycosylated) NEXT (hemoglobin* or haemoglobin*))):ti,ab,kw

12. #1 OR #2 OR #3 OR #4 OR #5 OR #6 OR #7 OR #8 OR #9 OR #10 OR #11

13. [mh Neoplasms] OR [mh “Stomach Neoplasms”]

14. ((gastric* OR stomach* OR gastrointestinal OR digestive) NEAR/3 (carcinoma* OR neoplasm* OR tumor* OR tumour* OR cancer* OR malignan*)):ti,ab,kw

15. #13 OR #14

16. ((neoplasm* OR tumor* OR tumour* OR cancer* OR malignan*) NEAR/3 (incidence OR screening OR detection OR risk* OR (risk NEXT assessment*) OR (risk NEXT factor*))):ti,ab,kw

17. [mh Risk] OR [mh “Risk Assessment”] OR [mh “Risk Factors”]

18. #16 OR #17

19. #12 AND #15 AND #18

| First Author and year | 1) Representativeness of the exposed cohort | 2) Selection of the non-exposed participants | 3) Loss of follow-up | Risk of selection bias based on item 1 to 3 | 4) Description of the fasting procedure | 5) Calibration of laboratory tests | 6) Demonstration that gastric cancer was not present at start of study | 7) Exclusion of prevalent cases of gastric cancer | 8) Outcome assessment | 9) Follow-up assessment | Risk of information bias based on item 4 to 9 | 10) Exclusion of known diabetes patients | 11) Key confounders  not controlled for | Risk of bias due to confounding based on item 10 and 11 | Over-all risk of bias |
| --- | --- | --- | --- | --- | --- | --- | --- | --- | --- | --- | --- | --- | --- | --- | --- |
| Travier 2007 | Somewhat representative | Yes | Unlikely | Moderate | N/A | No | Yes | No | Record linkage | No | Low | Yes | *H.p* infection^ξ^ , obesity | High | High |
| Ikeda 2009 | Truly representataive | Yes | Unlikely | Low | N/A | No | Yes | No | Multiple methods | Yes | Moderate | No | No | Low | Low |
| Goto 2016 | Truly representataive | Yes | Unlikely | Low | N/A | Yes | Yes | Yes | Record linkage | No | Low | Yes | *H.p* infection | Moderate | Low |
| Cheung 2019 | Truly representataive | Yes | Unlikely | Low | N/A | No | Yes | Yes | Record linkage | Yes | Low | Yes | Obesity | Low | Low |
| Peila 2020 | Somewhat representative | Yes | Unlikely | Moderate | N/A | No | Yes | Yes | Record linkage | Yes | Low | No | *H.p* infection | Moderate | High |
| Jee 2005 | Selected groups in the community | Yes | Unlikely | Low | Yes | Yes | Yes | No | Multiple methods | Yes | Low | Yes | *H.p* infection, obesity | High | High |
| Yamagata 2005 | Truly representataive | Yes | Unlikely | Low | Yes | No | Yes | Yes | Multiple methods | Yes | Moderate | No | No | Low | Low |
| Wulaningsih 2012 | Somewhat representative | Yes | Unlikely | Moderate | Yes | Yes | Yes | Yes | Record linkage | Yes | Low | No | *H.p* infection | Moderate | High |
| Lindkvist 2013 | No discrption | Yes | Unlikely | Moderate | Yes | No | Yes | Yes | Record linkage | Yes | Moderate | No | *H.p* infection | Moderate | High |
| Hidaka 2015 | Truly representataive | Yes | Unlikely | Low | Yes | Yes | Yes | No | Multiple methods | Yes | Moderate | No | No | Low | Low |
| Kim 2016 | Somewhat representative | Yes | No description | Moderate | Yes | No | Yes | No | Independent blind assessment | Yes | High | Yes | *H.p* infection, obesity | High | High |
| Pan 2018 | No discrption | Yes | No description | High | N/A | Yes | Yes | Yes | Multiple methods | Yes | High | Yes | *H.p* infection | Moderate | High |

**Part 2: Supplementary Table 1.** Assessment of risk of biases using a revised Newcastle-Ottawa Scale for cohort studies. Detailed assessment options for each item are listed below the table.

N/A: Not applicable

^ξ^ *Helicobacter pylori* infection

Assessment options for items 1 to 11 in the revised Newcastle-Ottawa Scale

1. **Representativeness of the exposed cohort:** (Truly representative/somewhat representative/selected group/no description)
2. **Selection of the non-exposed participants**: Are the non-exposed participants drawn from the same community as the exposed cohort? (Yes/No)
3. **Loss of follow-up:** The likelihood of bias introduced by loss of follow-up (Likely/unlikely/no description)
4. **Description of the fasting procedure**: Is there any description of how the fasting or glucose-load was performed? (Yes/No) *This item was evaluated only for studies reporting fasting or post-load glucose.*
5. **Calibration of laboratory tests:** (Yes/No)
6. **Demonstration that gastric cancer was not present at start of study:** (Yes/No)
7. **Exclusion of prevalent cases of gastric cancer:** (Yes/No)
8. **Outcome assessment:** The method how the outcome was assessed. (Independent blind assessment/record linkage/self-report/multiple methods/no description)
9. **Follow-up assessment:** Was follow-up long enough for outcomes to occur? (Yes if the average follow-up was more than 5 years/No)
10. **Exclusion of known diabetes patients:** Were patients with known diabetes excluded or analysed separately? (Yes/No)
11. **Key confounders not controlled for:** List any of the following potential important confounders that were not controlled for in the analyses; sex, age, *H.p* infection, and obesity.

**Supplementary Table 2.** Adjustment for co-variates in each included studies.

| **First Author and publication year** | **All adjusted co-variates** | **Adjustments for key co-variates** | | |
| --- | --- | --- | --- | --- |
|  |  | **Adjustment for *Helicobacter pylori (H. pylori)* infection** | **Adjustment for obesity** | **Adjustment for smoking habits** |
| Travier 2007 | Age, sex, ethnicity, smoking habits | No | No | Self-reported smoking habits (current smoker, ex-smoker, nonsmoker, and unknown status) |
| Ikeda 2009 | Age, sex, *H. pylori* infection, history of ulcer disease, alcohol intake, smoking habits, BMI, serum cholesterol levels, and dietary factors | Serum IgG antibodies to *H. pylori* infection (categorical: positive/ negative) | BMI calculated from objectively measured height and weight | Self-reported smoking habits (current user or not) |
| Goto 2016 | Age, sex, living area, BMI, smoking habits, physical activity, alcohol intake, dietary factors, and history of cardiovascular disease | No | BMI calculated from objectively measured height and weight | Self-reported smoking habits (current smoker, ex-smoker, and never smoker) |
| Cheung 2019 | Age, sex, *H. pylori* infection, smoking habits, alcohol intake, history of peptic ulcer disease, concurrent medication use, obesity, and other comorbidities | All participants were prescribed with a clarithromycin-based triple therapy for *H. pylori* infection | No | Documentation of the smoking status in the clinical data systems, the ICD-9 code of V15.82, or the presence of chronic obstructive pulmonary disease |
| Peila 2020 | Age, sex, ethnicity, education, socioeconomic status, smoking habits, pack-year of smoking, alcohol intake, physical activity, and BMI | No | BMI calculated from objectively measured height and weight | Self-reported smoking habits (current smoker, ex-smoker, and never smoker) and pack-year smoked for ever-smokers |
| Jee 2005 | Age, age squared, sex, smoking habits, alcohol intake | No | No | Self-reported smoking habits. Among ex-smokers and current smokers, amount smoked was adjusted for (1-9, 10-19, and >20 cigarettes/day). |
| Yamagata 2005 | Age, sex, BMI, serum cholesterol, *H. pylori* sero-positivity, smoking habits, alcohol intake, history of peptic ulcer disease, and dietary factors | Serum IgG antibodies to *H. pylori* infection (categorical: positive/ negative/indeterminate) | BMI calculated from objectively measured height and weight | Self-reported smoking habits (current user or not) |
| Wulaningsih 2012 | Age, gender, BMI, socioeconomic status, fasting status, total cholesterol (continuous), and triglycerides (continuous). | No | BMI calculated from objectively measured height and weight | No |
| Lindkvist 2013 | Age, sex, cohort, categorical birth year, BMI, fasting time, smoking habits. | No | Quintile levels of BMI. No information on how BMI was calculated | Yes, but unclear how this variable was adjusted for |
| Hidaka 2015 | Age, gender, area, BMI, blood donation date, fasting time, smoking habits, alcohol intake, dietary factors, *H. pylori* infection, gastric atrophy, family history of gastric cancer, history of diabetes, and diabetes treatment. | *H. pylori* infection positive: *H. pylori* antibody >10 U/ml or cytotoxin associated gene A (CagA) antibody >10; otherwise  *H. pylori* infection negative | BMI calculated from objectively measured height and weight (categorical: BMI <22, 22>=BMI<25, and 25<=BMI) | Self-reported smoking habits (never smoker, past smoker, current smoker with <=20 cigarettes per day, and current smoker with >=21 cigarettes per day) |
| Kim 2016 | Age, sex, smoking habits, alcohol intake, and total cholestrol levels | No | No | Self-reported smoking habits (current smoker, ex-smoker, and never smoker) |
| Pan 2018 | Sex, age, study area, education, BMI, smoking habits, alcohol intake, physical activity, and family histroy of cancer | No | BMI calculated from objectively measured height and weight | Yes, but unclear how this variable was adjusted for |

**Supplementary Figure 1.** Forest plot of risk estimates for associations between different levels of serum haemoglobin A1c and risk of gastric cancer under random-effects model. CI: Confidence interval

**
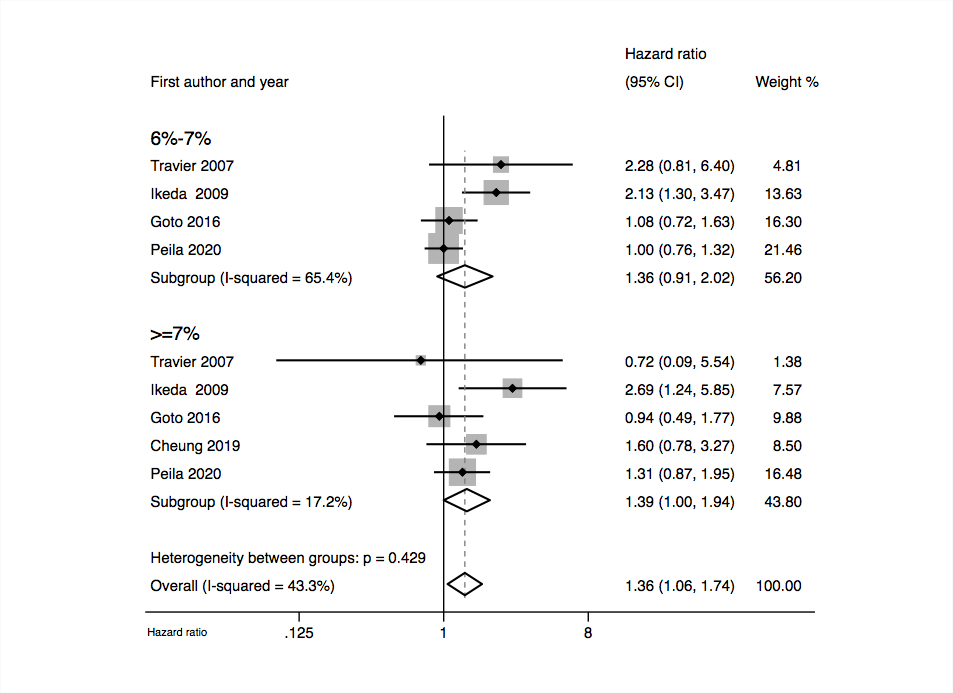
**

**Supplementary Figure 2.** Forest plot of risk estimates for associations between different levels of fasting serum glucose and risk of gastric cancer under random-effects model. CI: Confidence interval


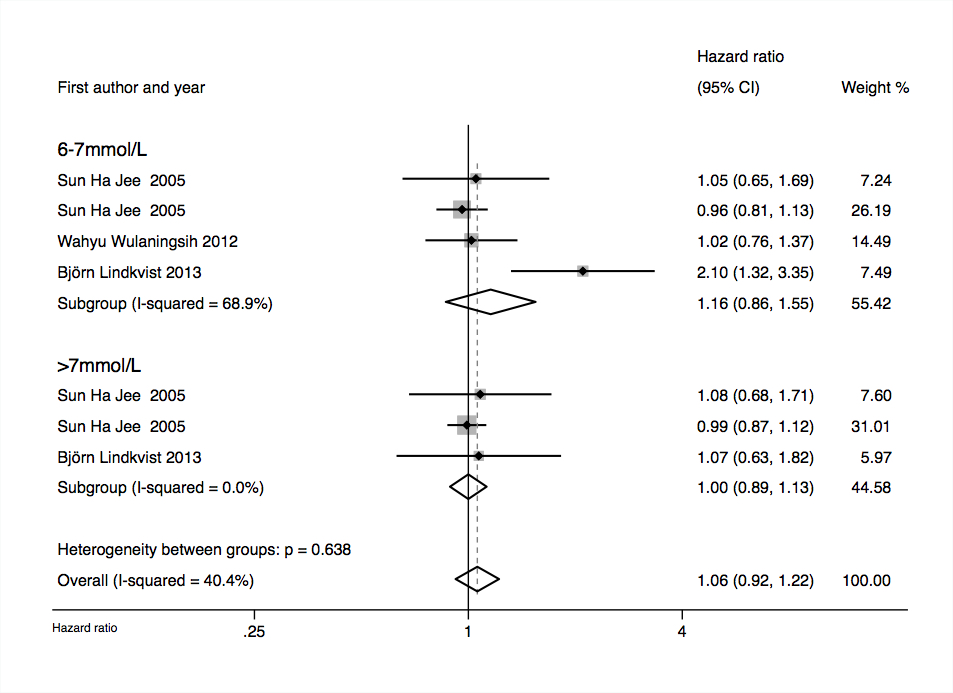

Supplement: Supplementary file 1 — Supplementary Material [file 41416_2021_1693_MOESM1_ESM.docx]
